# Supplementary material for: MetaRibo-Seq measures translation in microbiomes
Source: Nat Commun. 2020 Jun 29;11:3268. doi: 10.1038/s41467-020-17081-z (PMC7324362; doi:10.1038/s41467-020-17081-z)
Supplement: Supplementary file 10 — Supplementary Data 7 [file 41467_2020_17081_MOESM10_ESM.zip › File2/Confidence_VeryHigh_Taxonomy/687_out.krona.html]

Javascript must be enabled to view this page.

members
magnitude
magnitudeUnassigned
count
unassigned
taxon
rank

687\_out

12

2
12
superkingdom

phylum
12
1239

186801
class
12

186802
order
12

9
family
541000

1952411

SRS148511\_contig\_number\_14571
1
species

8
genus
1263

1
species
46228

SRS019161\_contig\_number\_34912

7
species
165186

SRS016095\_contig\_number\_20764SRS019496\_contig\_number\_contig-100\_1675.77294SRS055982\_contig\_number\_18154SRS062654\_contig\_number\_14166SRS149181\_contig\_number\_2673SRS893170\_contig\_number\_contig-100\_31341.31342SRS893270\_contig\_number\_2339

186803
1
2
family

SRS014613\_contig\_number\_contig-100\_24933.24933

841
1
genus

1
species
360807

SRS015217\_contig\_number\_contig-100\_706.189633

216572
1
family

1
genus
459786


SRS011134\_contig\_number\_43893
1945593
species
1
